# Molecular dynamics simulation of aluminium binding to amyloid-β and its effect on peptide structure

**DOI:** 10.1371/journal.pone.0217992

**Published:** 2019-06-11

**Authors:** Matthew Turner, Shaun T. Mutter, Oliver D. Kennedy-Britten, James A. Platts

**Affiliations:** School of Chemistry, Cardiff University, Park Place, Cardiff, United Kingdom; Russian Academy of Medical Sciences, RUSSIAN FEDERATION

## Abstract

Multiple microsecond-length molecular dynamics simulations of complexes of Al(III) with amyloid-β (Aβ) peptides of varying length are reported, employing a non-bonded model of Al-coordination to the peptide, which is modelled using the AMBER ff14SB forcefield. Individual simulations reach equilibrium within 100 to 400 ns, as determined by root mean square deviations, leading to between 2.1 and 2.7 μs of equilibrated data. These reveal a compact set of configurations, with radius of gyration similar to that of the metal free peptide but larger than complexes with Cu, Fe and Zn. Strong coordination through acidic residues Glu3, Asp7 and Glu11 is maintained throughout all trajectories, leading to average coordination numbers of approximately 4 to 5. Helical conformations predominate, particularly in the longer Al-Aβ40 and Al-Aβ42 peptides, while β-strand forms are rare. Binding of the small, highly charged Al(III) ion to acidic residues in the N-terminus strongly disrupts their ability to engage in salt bridges, whereas residues outside the metal binding region engage in salt bridges to similar extent to the metal-free peptide, including the Asp23-Lys28 bridge known to be important for formation of fibrils. High helical content and disruption of salt bridges leads to characteristic tertiary structure, as shown by heat maps of contact between residues as well as representative clusters of trajectories.

## Introduction

Alzheimer's disease (AD) is a devastating neurodegenerative condition that poses major healthcare challenges. Significant hallmarks of AD include the death of neurons and their connections in addition to the presence of insoluble plaques and neurofibrillary tangles. The amyloid hypothesis suggests that aggregation of the amyloid-β (Aβ) peptide into soluble oligomers and senile plaques is the main driver of AD.[1,2] In contrast, the metal ion hypothesis suggests that disruption of metal ion homeostasis promotes Aβ aggregation and onset of AD.[3–5] The primary focus of the metal ion hypothesis has been on naturally occurring metals, particularly Cu(II),[6–11] Zn(II),[12–15] and Fe(II),[16–20] though studies over several decades have also linked aluminium(III) with the development of AD.[21–25] Aluminium does not naturally occur in human biology, but Al(III) is a known neurotoxin that interacts with a range of metal-binding proteins, influencing the homeostasis of other ions. Specifically, Exley et al showed that aluminium induces conformational changes in Aβ,[26] that have been linked to inhibition of Aβ degradation as well as promotion of aggregation,[27] and formation of reactive oxygen species.[28]

To date, several simulation studies on the interaction of aluminium with Aβ have been reported, focussing on the binding modes of Al(III) with Aβ and the resulting morphology of Al-Aβ aggregates.[29,30] Recently, Mujika et al used a computational approach to generate 3D models of Al(III) bound to the N-terminal Aβ16 fragment of Aβ in a 1:1 stoichiometry.[31] Here, Al shows a clear preference for coordination by carboxylate groups (Glu3, Asp7 and Glu11) with the remaining coordination sites occupied by backbone carbonyl moieties or water molecules. In addition, the authors performed molecular dynamics (MD) simulations of the resulting complexes. These validated their identified coordination modes, but were too short to properly sample the ensemble of structures available to such complexes due to the inherently flexible and disordered nature of Aβ. In this context, we note that a recent study has compared the effect of Li, Na and K ions on Aβ structure and stability.[32]

In order to examine Al-Aβ interactions in more detail, as well as their effect on peptide structure, we report MD simulations of Al(III) interacting with the N-terminal AΒ16 fragment as well as the full Aβ40 and Aβ42 peptides, using molecular mechanics incorporating a non-bonded model of Al(III) coordination. In this way, we aim to sample a wide range of conformations of the intrinsically disordered Aβ peptide as well as allowing Al(III) to sample a range of coordination sites in the peptide.

## Computational methods

Aβ peptides were constructed in extended conformations in MOE[33] with appropriate residue protonation states for physiological pH. Al(III) was coordinated via Glu3, Asp7 and Glu11, as identified by Mujika et al.[31] Structures were subjected to short LowMode[34] conformational searches to obtain reasonable starting structures. MD simulations were performed using the AMBER16 package.[35] The AMBER ff14SB forcefield parameter set[36] was used to model all standard amino acid residues; the non-bonded MCPB.py[37] approach was used for Al(III), enabling the metal ion to sample different coordination sites during simulation. RESP charges for the metal-coordinating regions were obtained from B3LYP/6-31G(d) calculations using Gaussian09.[38] This combination of functional and basis set matches that used by Li and Merz in developing and testing MCPB.py, and is recommended for compatibility with AMBER-style forcefields. The Al(III) ion was fixed as a 3+ charge, with radius of 1.37 Å.

The Generalised Born solvation model was used to solvate all Al(III)-Aβ systems,[39] as this has been shown to enhance sampling of flexible systems.[40] MD simulations were carried out in the NVT ensemble, using the Langevin thermostat to control temperature. The SHAKE algorithm was used to constrain bonds to hydrogen, and simulations performed using a 2 fs integration timestep. Analysis of the trajectories was performed using CPPTRAJ v16.16[41] and VMD 1.9.3.[42] Secondary structure assignment was made using the DSSP algorithm[43] in CPPTRAJ. Salt bridges were defined as any contact distance of less than 3.2 Å between oxygen and nitrogen atoms in charged residues. Clustering used the DBSCAN algorithm of Ester et al.[44] Ramachandran maps and hydrogen bonding plots were made using MDplot.[45]

## Results

**Fig 1:**
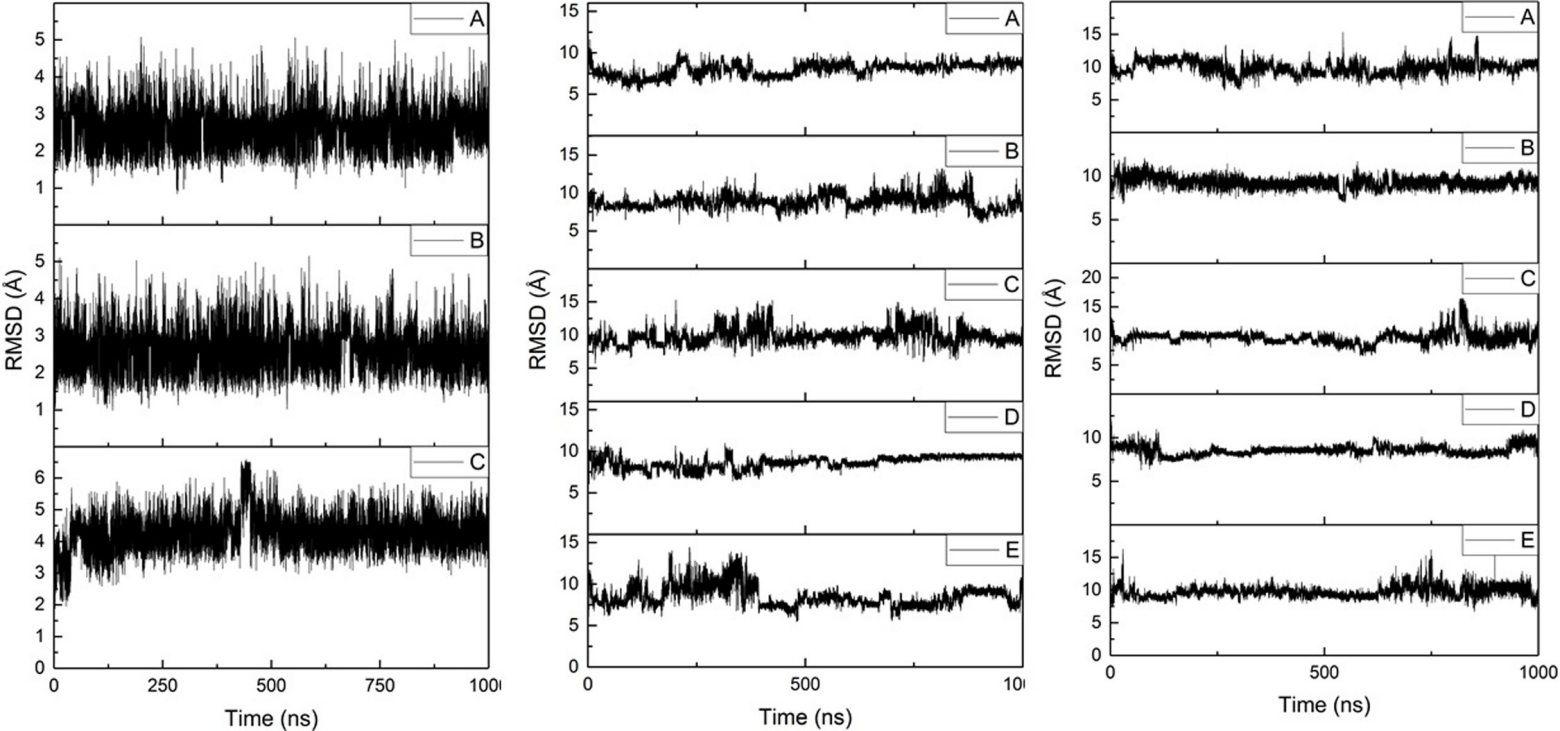
Backbone RMSD for simulations of Al-Aβ16, Al-Aβ40 and Al-Aβ42.

Three separate 1 μs simulations of Al-Aβ16 (denoted A, B and C) were performed, starting from the same minimised conformation, but with different random velocities sampled from the Maxwell-Boltzmann distribution at 310 K. Similarly, five 1 μs simulations (A-E) were performed for both Al-Aβ40 and Al-Aβ42. Root mean square displacement (RMSD) relative to the starting structures was used as a measure of equilibration.[9,46] Fig 1 shows RMSD plots for all backbone atoms in Al-Aβ16, Al-Aβ40 and Al-Aβ42 systems, respectively, relative to their respective minimised structures. Equilibration points are shown in Table 1; Al-Aβ16 simulations reach stable values rapidly, while Al-Aβ40 and Al-Aβ42 simulations take longer to equilibrate. All analysis reported is taken from data extracted from frames after each equilibration point, averaged over the relevant simulations, such that trajectories of 2.7, 2.1 and 2.4 μs are available for further analysis. Averages and standard deviations of RMSD are shown in Table 2, further illustrating the equilibration of the trajectories: standard deviations of around 1 Å are found across the multiple microsecond trajectories. As might be expected, the larger peptides reach larger average values of RMSD than the N-terminal fragment, with a notable increase for the 42-residue peptide over the 40-residue one.

**Table 1:** Equilibration point for simulations (ns).

	Al-Aβ16	Al-Aβ40	Al-Aβ42
A	100	480	320
B	100	50	200
C	125	400	100
D		100	240
E		400	200

**Table 2:** RMSD statistics for post-equilibration simulations (Å).

	Ave	SD	Min	Max
Aβ16	3.19	0.93	0.85	6.59
Aβ40	8.78	1.15	1.91	15.26
Aβ42	9.43	0.91	6.52	16.46

In principle, the non-bonded model of Al(III) coordination used allows the ion to sample alternative donor atoms than the carboxylate sidechains at which simulations were started. All Al—O distances were monitored over all equilibrated frames for Al-Aβ42, from which it is clear that the anionic Glu3, Asp7 and Glu11 residues dominate binding. Distances of 1.858 ± 0.036 Å for Glu3 sidechain O, 1.813 ± 0.030 Å for Asp7 sidechain O, and 2.615 ± 0.865 Å and 2.917 ± 0.887 Å for each carboxylate O in Glu11 sidechain are recorded. This indicates that mono-dentate binding prevails for Glu3 and Asp7, whereas Glu11 binds more in bi-dentate fashion. Transient Al—O contacts are also observed for backbone O of Glu3 (minimum 1.769, mean 4.440 Å) and Phe4 (minimum 3.313, mean 7.457 Å) are also observed.

**Fig 2:**
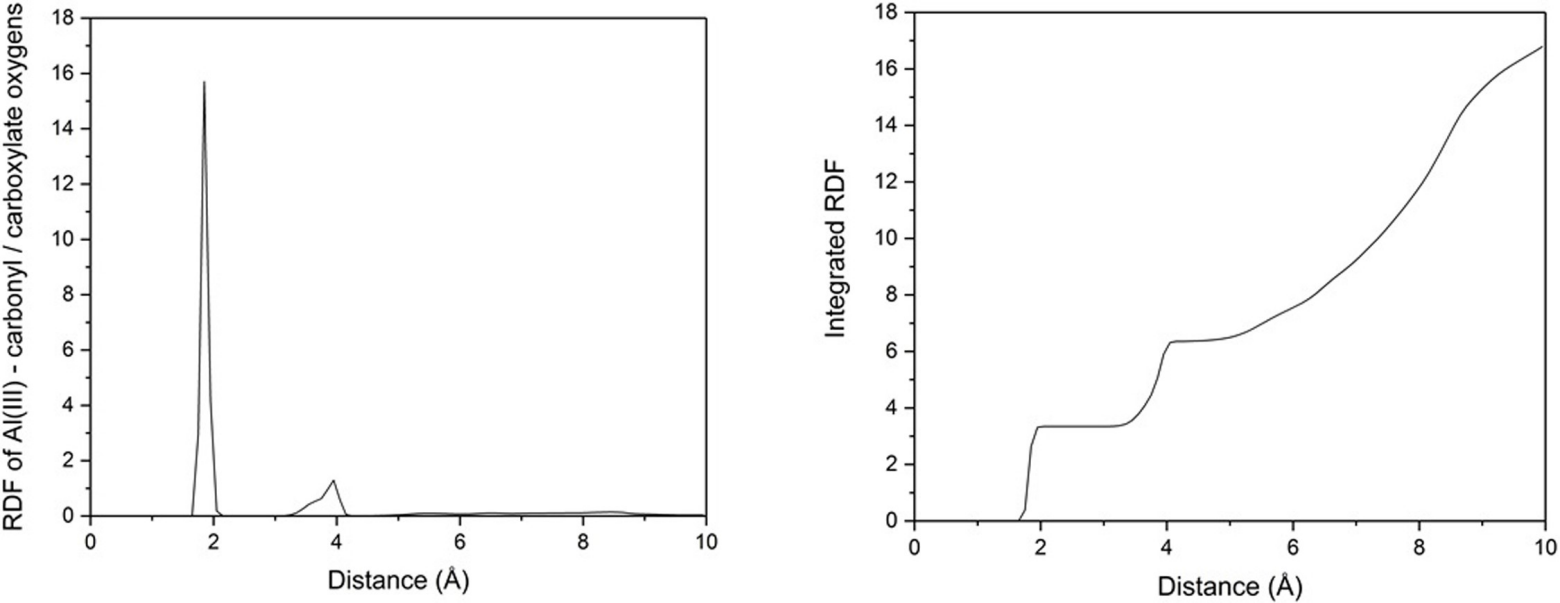
(a) RDF and (b) integrated RDF of Al-oxygen distances in Al-Aβ42.

Summing across all oxygen atoms for all frames in the equilibrated Al-Aβ42 trajectory leads to the radial distribution function (RDF) for ion coordination, as shown in Fig 2A. RDFs for smaller peptides (reported in ESI) are similar. A single sharp peak centred on 1.9 Å corresponds to dominant sidechain coordination, while a lower and broader feature around 3.8 Å shows evidence for some outer-sphere interaction. Integrating the RDF (Fig 2B) leads to an average coordination number of 3.7 for Al.

**Fig 3:**
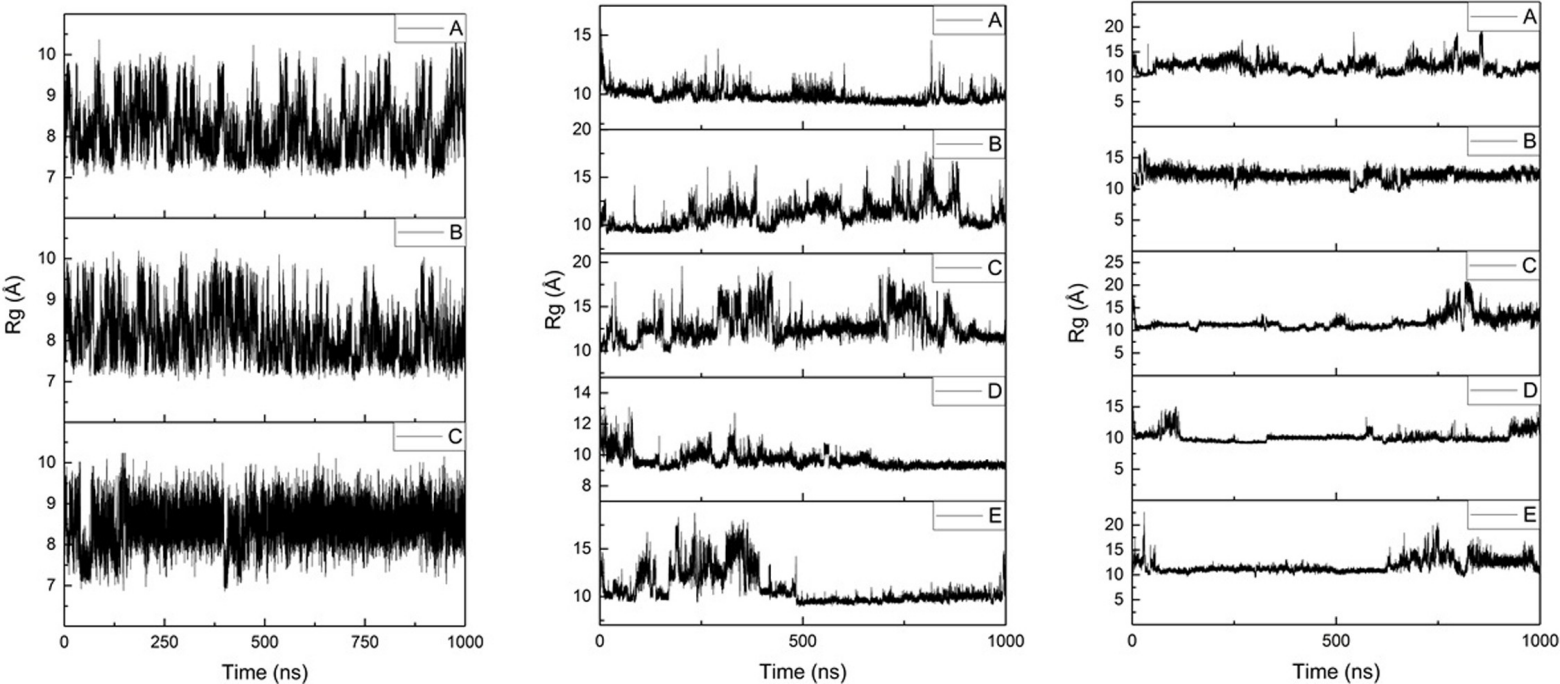
Rg plots for Al-Aβ16, Al-Aβ40 and Al-Aβ42 simulations.

Radius of gyration (Rg) values show similar trends to RMSD; plots of Rg as a function of simulation time are shown in Fig 3 and summary statistics in Table 3. Al-Aβ16 displays the smallest average Rg (*ca*. 8 Å) among the systems studied, while the much longer Al-Aβ40 and Al-Aβ42 peptides exhibit only slightly larger average Rg values (*ca*. 10.6 and 11.6 Å, respectively), suggesting that the larger peptides adopt collapsed rather than extended conformations in the presence of Al(III). For comparison, Rg values of 13.1 Å (using AMBER ff03) and 10.1 Å (using AMBER ff99SB) have been reported in literature for the metal-free Aβ42.[9,47] Rg values for Cu, Fe and Zn bound to Aβ16 and sampled in similar manner[48] were 7.6, 7.2 and 8.1 Å, respectively, indicating that Al(III) coordination leads to less compact structures than do those metals.

**Table 3:** Rg statistics for post-equilibration simulations (Å).

	Ave	SD	Min	Max
Aβ16	8.24	0.59	6.86	10.30
Aβ40	10.59	1.52	8.89	19.44
Aβ42	11.58	1.41	8.97	20.92

**Fig 4:**
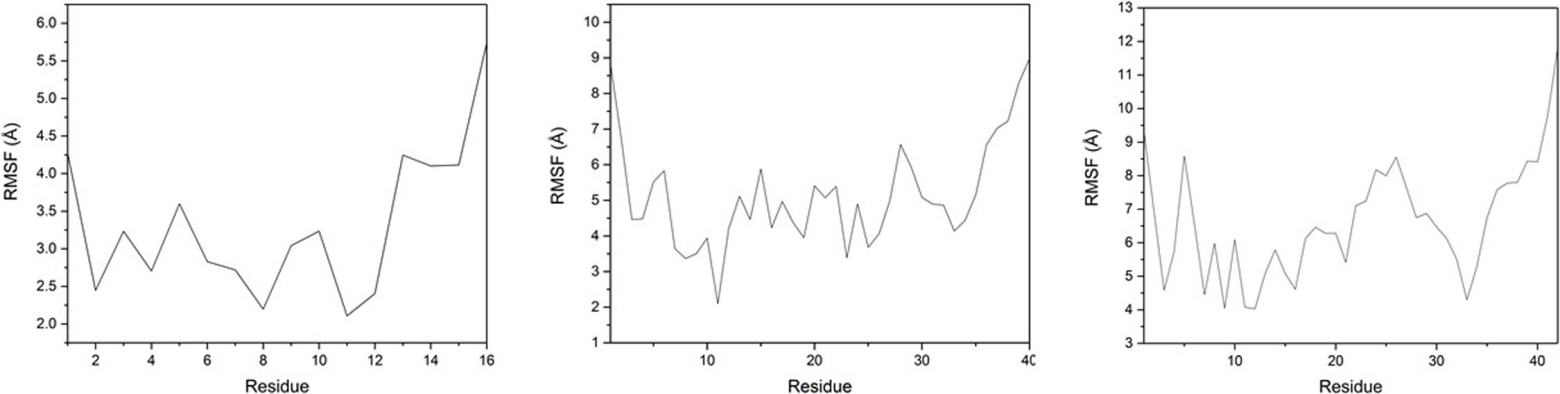
RMSF plots, by residue, for Al-Aβ16, Al-Aβ40 and Al-Aβ42.

Root-mean-square fluctuation (RMSF) values for individual residues are shown in Fig 4, with associated data in Supporting Information. For Al-Aβ16, both termini (Asp1 and His13-Lys16) are flexible with RMSF values > 4.0 Å, whereas Ala2, Ser8, Glu11 and Val12 are the least flexible residues (RMSF < 2.5 Å). Interestingly, the metal binding residues Glu3 and Asp7 exhibit greater fluctuation than their neighbours, in contrast to previous work using an explicit metal-ligand bonding terms within the molecular mechanics energy expression.[48] In the larger peptide systems, different patterns of residue flexibility are observed: in both cases, Glu11 stands out as particularly stable, while other coordinating residues are also relatively inflexible. The anchoring effect of coordination extends beyond individual residues to encompass the whole of the Asp7-Glu11 segment (RMSF < 3.6 Å), whereas Arg5 is highly mobile in both cases. In addition, residues corresponding to the central hydrophobic core (CHC, *i*.*e*. Leu17 to Ala21) and the C-terminus are found to undergo large fluctuations, reflecting the expected unstructured nature of the single peptide in aqueous conditions. In both cases, Gly33 has relatively low RMSF: as a small residue close to the C-terminus, this might be expected to highly mobile. Further analysis (*vide infra*) indicates this residue is involved in hydrogen bonding. The final two residues of Aβ42 are particularly mobile, indicating a lack of structure of the C-terminus in the larger peptide and contributing to the larger overall RMSD noted above.

**Fig 5:**
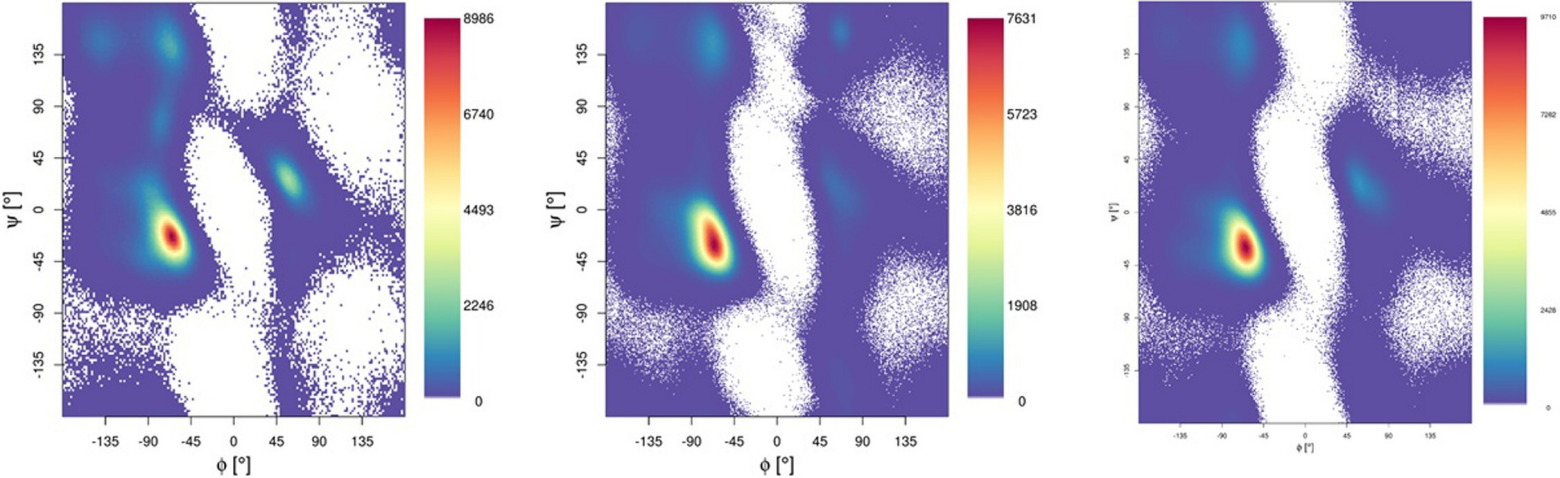
Ramachandran plots for simulations of Al-Aβ16, Al-Aβ40 and Al-Aβ42.

**Fig 6:**
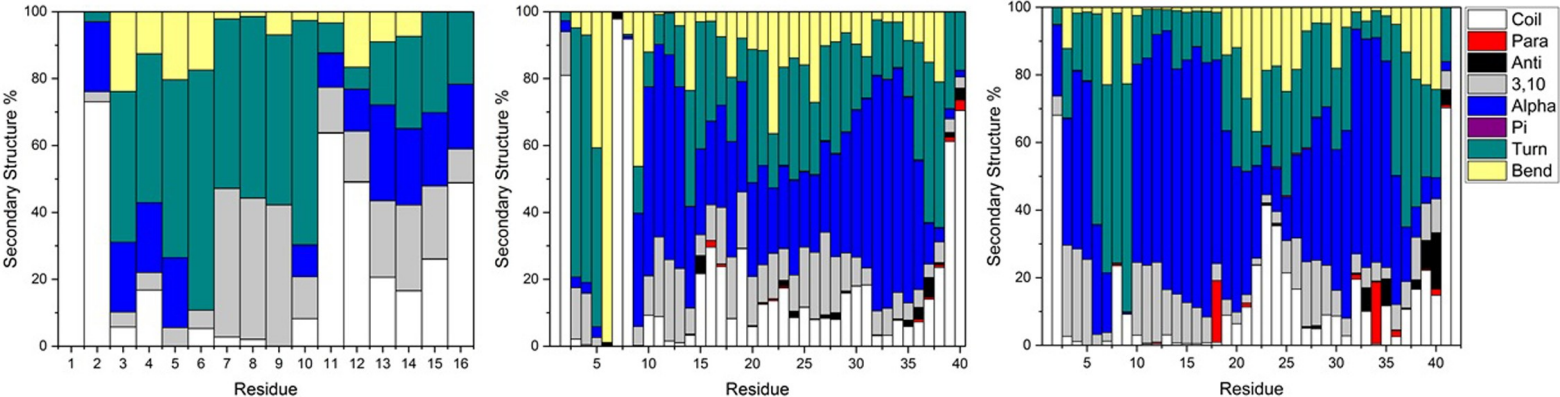
Peptide secondary structure percentage, by residue, for Al-Aβ16, Al-Aβ40 and Al-Aβ42.

Secondary structure adopted by Aβ, and the effect of Al-binding on this, is a key aspect of the metal hypothesis for AD. Fig 5 shows the Ramachandran maps that result from post-equilibration MD trajectories for all three peptides bound to Al(III), showing that the peptides spend most time in the general region of α-helices, with smaller but noticeable populations of β-strand, poly-proline II and left-handed helix regions and a rather broad distribution in *ϕ*/Ψ space. The relative significance of the non- α-helix regions diminishes in the larger peptides compared to Al-Aβ16. These observations can be quantified by assignment of secondary structure using DSSP, as summarised in Fig 6. This shows that coil/turn/bend structures predominate in the N-terminal region, along with a smaller contribution from 3,10 and α-helices. Key metal-binding residues 3, 7 and 11 do not have noticeably different secondary structure from the rest of the N-terminus, nor from previous reports for metal-free Aβ42. Helical content increases beyond the immediate N-terminus, although significant amounts of 3,10 and α-helix are apparent in Glu11-Lys16 in all systems. The CHC exhibits relatively few helical residues, and also a small amount of β-strand, which is mostly antiparallel in Al-Aβ40 but parallel in Al-Aβ42. Helical content then increases toward the C-terminus, reaching maximal values in the sequence Ala30 to Val36, while the final few residues of both peptides are rather unstructured. Values summed across the entire peptides are reported in Table 4, and show increasing amounts of helical content as the peptide elongates. Even for Al-Aβ16, 30% of residues are classified as helical, rising to almost 40% of Al-Aβ40 and nearly 50% of Al-Aβ42. Fig 6, along with visualisation of representative clusters of trajectories (*vide infra*), shows that this helical character does not take the form of a single helix, but rather of several short (4 to 8 residue) segments with helical form.

**Table 4:** Total secondary structure character for Al-Aβ16, Al-Aβ40 and Al-Aβ42.

	Helix	Sheet	Other
Aβ16	30.2	0.0	69.8
Aβ40	39.4	1.1	59.4
Aβ42	48.2	2.3	49.5

**Fig 7:**
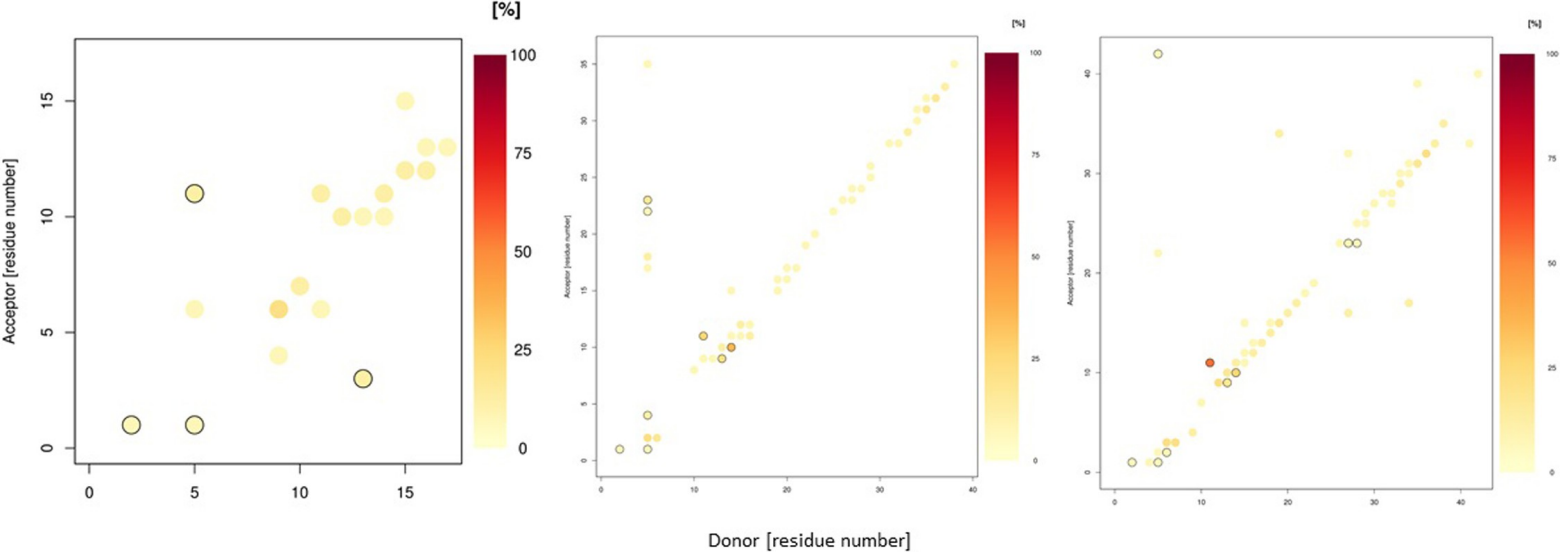
Hydrogen bond incidence plots for Al-Aβ16, Al-Aβ40 and Al-Aβ42 simulations. Black circles indicate more than one type of hydrogen bond between the relevant residues.

Table 5 shows statistics relating to the number of hydrogen bonds present over equilibrated simulations. Aβ16 has approximately four hydrogen bonds per simulation frame, although the standard deviation value suggests that these interactions are transitory; this system also contains frames with as many as 14, or as few as zero hydrogen bond interactions. Aβ40 contains, on average, just less than twelve hydrogen bonds per frame: again this covers great variability with as many as 26 simultaneous hydrogen bonds, as well as frames with none. Aβ42 contains almost 14 hydrogen bonds per simulation frame, on average, with as few as 2 or as many as 32 hydrogen bonds present in a given frame. The specific identities and prevalence of these interactions are shown in Fig 7. In Al-Aβ16, there are 471 different hydrogen bond interactions present for at least 1 frame; the most common (reported as donor-acceptor) are between Gly9 backbone-His6 backbone (20.7%), His14 sidechain-Glu11 backbone (14.0%) and Gln15 backbone-Val12 backbone (13.2%). There are twelve i+4 -> i backbone-backbone hydrogen bonds, characteristic of α-helices, though only three are present for more than 5% of simulation. Similarly, there are 13 i+3 -> i hydrogen bonds (typical of 3,10-helices) and 8 i+5 -> i interactions (π-helices), though only 4 and 2, respectively, are present for 5% or more of simulation time.

**Table 5:** Hydrogen bond statistics for Al-Aβ16, Al-Aβ40 and Al-Aβ42 simulations.

	Ave	SD	Min	Max
Aβ16	4.13	1.79	0	14
Aβ40	11.96	3.04	0	26
Aβ42	13.70	3.44	2	32

In Al-Aβ40 there are 1807 different hydrogen bonds observed, the most prevalent being His14 sidechain-Tyr10 backbone (33.4%), Glu11 backbone-Glu11 sidechain (28.1%) and His13 sidechain-Gly9 backbone (20.9%). Of these, 32 are i+4 -> i backbone-backbone hydrogen bonds, of which 15 are present for more than 5% of simulation; notable contributions are made by Ile32-Val36 (18.9%) and Ile31-Met35 (15.2%). Furthermore, there are 14 i+3 -> i and just 1 i+5 -> i interactions present in excess of 5% of simulation, reflecting the greater contribution of α-helices. The hydrogen bonding pattern is similar Aβ42, common contacts include Glu11 backbone-Glu11 sidechain (53.1%), His14 sidechain-Tyr10 backbone (27.4%) and Asp7 backbone-Glu3 backbone (22.4%). In total, 1688 hydrogen bonds are observed; 19 i+4 -> i hydrogen bonds, 16 i+3- > i hydrogen bonds and 2 i+5 -> i hydrogen bonds each over 5% occupancy.

**Fig 8:**
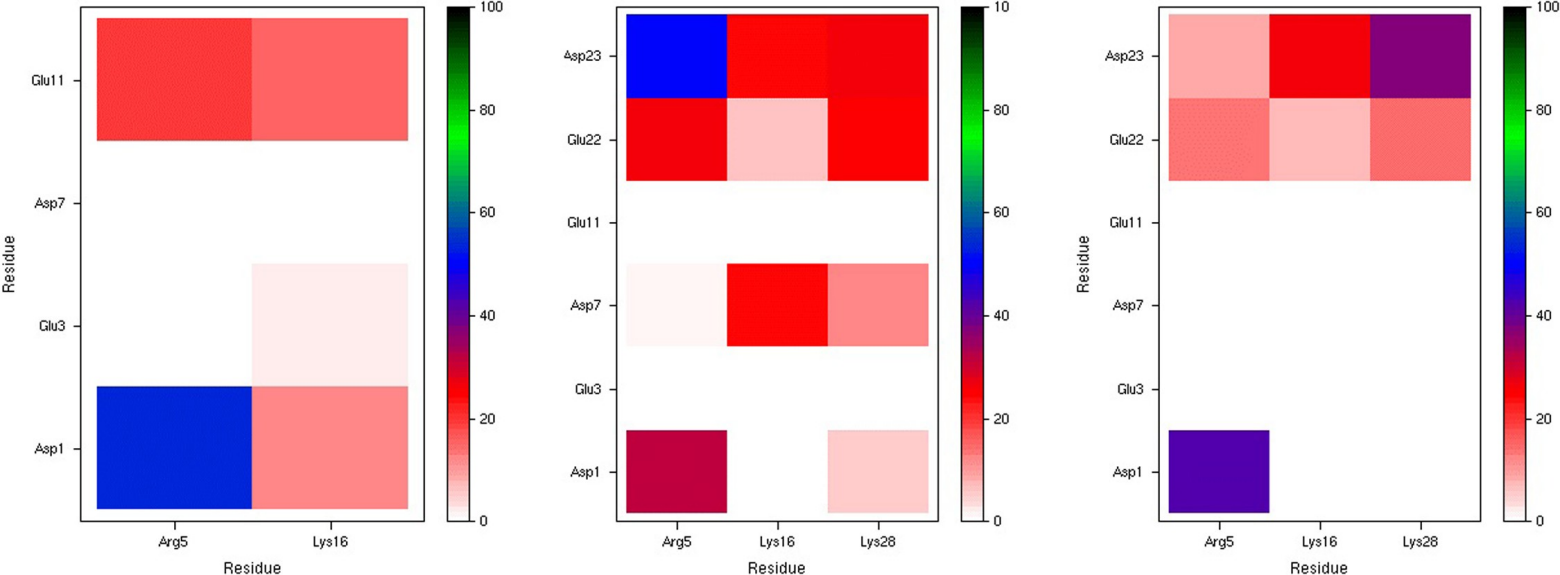
Salt bridge incidence plots (%) for Al-Aβ16, Al-Aβ40 and Al-Aβ42 simulations.

Salt bridge interactions also play an important role in the stability of amyloid-β structures. The incidence of these interactions is summarised in Fig 8. In Aβ16, there are eight possible salt bridges, five of which are occupied for at least 1% of total simulation time. Of these, the most common are Asp1-Arg5 (54%), followed by Glu11-Arg5 (20%). Interestingly, Asp1 and Glu11 form more salt bridges than Glu3 and Asp7. This may be because the more central Glu3 and Asp7 residues are commonly involved in coordination of Al, such that their charged sidechains are less available for salt bridge formation. In Aβ40 and Aβ42, there are eighteen possible salt bridges: Aβ40 has the greater range of salt bridge interactions, with eleven present for at least 1% of simulation time. The most common is Asp23-Arg5 (51%), with Asp1-Arg5 interaction also frequently observed (32%). Asp7 also forms numerous salt bridges (with Lys16: 24%, Lys28: 12%). However, the Aβ42 salt bridge network is notably different from Al-Aβ40; just seven of the eighteen possible interactions are present. Asp1-Arg5 is the most frequent interaction (43%), while Asp23-Lys28 is also prevalent (37%), but here Asp7 is not involved in any bridges.

**Fig 9:**
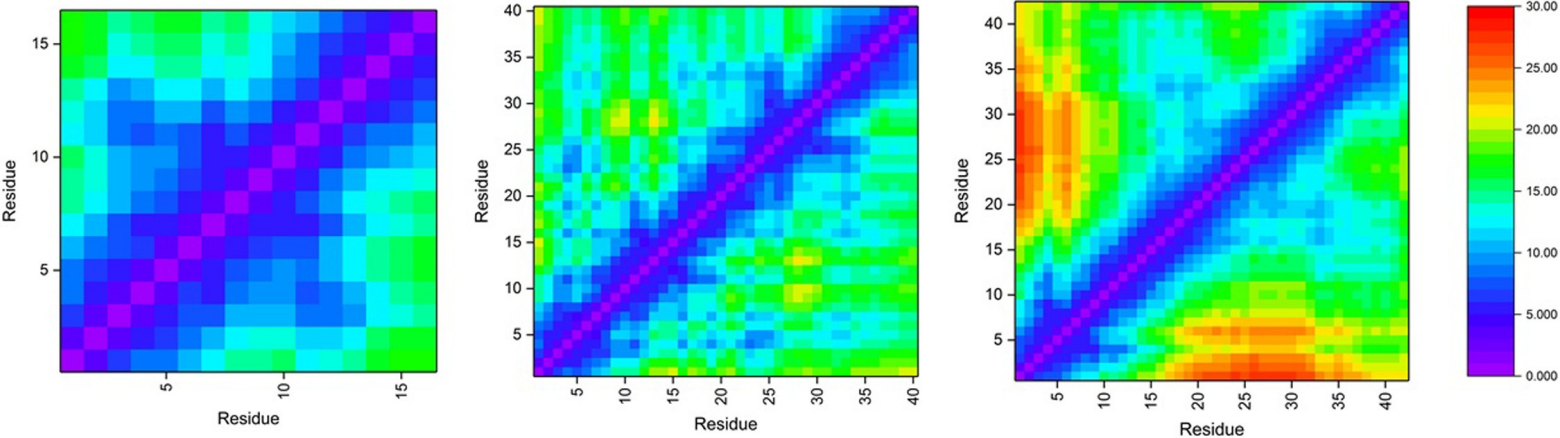
Cα–Cα contact maps for Al-Aβ16, Al-Aβ40 and Al-Aβ42.

Tertiary structure of the Al-Aβ complexes are summarised using the Cα-Cα contact maps, as shown in Fig 9. Al-Aβ16 exhibits short contacts between Glu3-Phe4 and Glu11-His13, apparently driven by the constraints of metal coordination as well as the Arg5-Glu11 salt bridge. The same pattern is also present in Al-Aβ40 and Al-Aβ42. Al-Aβ40 also exhibits close contacts between Phe4-Arg5 and Glu22-Gly25, due to the prevalence of salt bridges to Glu22 and Asp23 in this system. The reduced incidence of these salt bridges in the longer Al-Aβ42 means that short contacts of the N-terminus with the central are much less evident here. Both longer peptides also have notable short contacts of Gly33 with Lys16-Ala21, *i*.*e*. central hydrophobic cluster, giving rise to the low RMSF observed for this residue.

**Fig 10:**
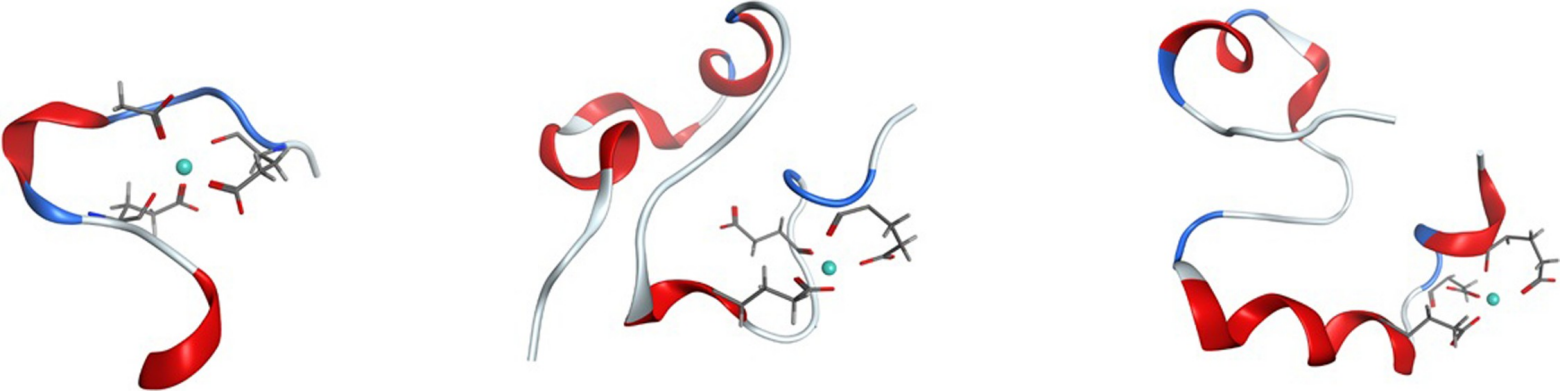
Most populated clusters for Al-Aβ16, Al-Aβ40 and Al-Aβ42 simulations. Al(III) is shown as a cyan sphere, selected atoms of Glu3, Asp7 and Glu11 as wireframe, and the remainder of the peptide as a ribbon, coloured by secondary structure (red = helix, blue = turn, white = coil).

Clusters were extracted from equilibrated trajectories: for Al-Aβ16 the most populated cluster is present for 25.4% of frames. As shown in Fig 10, this cluster is made up of coil in the N-terminal region, a turn centred on Gly9, and a helical section at the C-terminus, with Al(III) bound through backbone and sidechain O of Glu3 and Glu11 as well as sidechain of Asp7. Small elements of helical content are apparent between Asp7 and Glu11, and also at the C-terminal Lys16. The same process leads to less populated clusters for the longer peptides, indicating their more unstructured nature: the most populated clusters for Al-Aβ40 and Al-Aβ42 occupy just 4.1% and 2.5% of simulation time, with no other cluster accounting for as much as 1%. In both cases, a relatively unstructured N-terminus binds Al(III) through acidic sidechains. The Al-Aβ40 cluster is quite unstructured over the whole sequence, with small elements of helical structure in both the CHC and C-terminus, but neither extending over more than a handful of residues. In contrast, Al-Aβ42 includes an α-helix over Glu11-Phe20, followed by a turn centred on Ala21 and a relatively unstructured C-terminus.

## Discussion

Microsecond timescale simulations of Al(III) bound to Aβ16, Aβ40 and Aβ42 in implicit aqueous solvent reveal a picture of a flexible, unstructured set of systems in which no one structure dominates. Equilibration, as judged by time evolution of RMSD, is fast for the smallest peptide, but takes several hundred nanoseconds for the larger ones. The non-bonded model of ion coordination employed allows the ion to sample numerous ligating sites within the peptide, but in fact we find that coordination to the N-terminal acidic residues Glu3, Asp7 and Glu11 is highly stable across over 2 μs of MD trajectories for each system. This leads to average coordination number in the inner coordination shell for Al of around 4, with a further 2 to 3 oxygens in the outer coordination shell.

Al-Aβ16 is a useful, computationally tractable model for metal coordination, but termination just beyond the metal binding N-terminus means that some properties are notably different between this and the full-length peptides. Nevertheless, some observations can be made for all three systems, in particular the relative lack of helical/sheet secondary structure in the N-terminus, low RMSF for metal-binding residues Glu3, Asp7 and Glu11, and the high occupation of the Asp1-Arg5 salt bridge. In contrast, salt bridge patterns beyond the N-terminus as well as the tertiary structure differ between Al-Aβ16 and the longer peptides.

Comparing Al-Aβ40 with Al-Aβ42 is instructive. The latter equilibrates to larger RMSD and Rg values, and the final two residues (Ile41 and Ala42) are particularly mobile in RMSF measurements. These residues adopt little secondary structure, Ala42 in particularly remaining classified as coil throughout all trajectories, while Ile41 includes small amounts of turn, helical and sheet types. The presence of these unstructured, hydrophobic amino acids affects the pattern of salt bridges: Asp23-Lys28, known to be important in the conformational changes that accompany formation of fibrils,[49] is favoured over Asp23-Arg5, seen in Al-Aβ40. The flexible, hydrophobic residues and changed salt bridge pattern are reflected in the tertiary structures of Aβ40 and Aβ42, the latter adopting a more expanded set of conformations with particularly long distances between N-terminal residues and those between Phe20 and Ala30.

It also interesting to compare the trajectories reported here with those for the free Aβ42 and its adducts with Cu(II) and Pt(II)-phenanthroline, as reported by us recently.[48] Al(III) leads to structures that are larger than the free peptide (Rg = 9.6 Å) but smaller than Cu (13.8 Å) or Pt (16.5 Å) adducts, perhaps reflecting the harder nature of the small, highly charged Al(III) ion. Secondary structure, contact maps and salt bridge patterns are most similar to those observed for Cu(II) coordination, despite the fact that this ion binds through different residues (Ala2, His6, His13 and His14), suggesting that the identity of the metal ion and/or coordinating residues are less important than the presence/absence of a ligand free ion bound to the N-terminal region of Aβ42.

## Conclusions

Our data show that atomistic molecular dynamics simulations of Al(III) bound to amyloid-β peptides of different lengths, using a non-bonded model of ion coordination, equilibrate in timescales of several hundred nanoseconds. Collating post-equilibration trajectories from multiple microsecond MD runs indicates stable binding of Al(III) to acidic residues in the N-terminal region of Aβ and an average coordination number of around 4. The metal binding residues Glu3, Asp7 and Glu11 are typically relatively immobile, while N- and C-terminal residues are highly flexible. This flexibility still allows significant quantities of secondary structure to develop: the longest peptide, Aβ42, reaches almost 50% helical content, this being concentrated largely in residues 11–20 and 26–36. Salt bridges are strongly affected by the presence of the ion, most notably as coordination to acidic N-terminal residues severely retards their ability to form salt bridges. This, along with hydrogen bond patterns that reflect the rather high helical content, leads to characteristic patterns in tertiary structure in which stable contacts between salt bridged pairs, as well as residues bound to Al(III) are apparent. Overall, we find that coordination of Al(III) to the N-terminus of Aβ has a major impact in the structure and dynamics of the peptide, inducing significant helical content, reducing the impact of salt bridges, reducing the flexibility of binding residues and increasing that of terminal residues.

## Supporting information

S1 Fig RDF and integrated RDF of Al-oxygen distances in Al-Aβ16. (DOCX)

S2 Fig RDF and integrated RDF of Al-oxygen distances in Al-Aβ40. (DOCX)

S1 Table RMSD data for individual MD runs. (DOCX)

S2 Table RMSF by residue. (DOCX)

S3 Table Salt bridge incidences (%). (DOCX)
