# Supplementary figures and images for: Molecular dynamics simulation of aluminium binding to amyloid-β and its effect on peptide structure
Source: PLoS One. 2019 Jun 11;14(6):e0217992. doi: 10.1371/journal.pone.0217992 (PMC6559712; doi:10.1371/journal.pone.0217992)

S1 Figure: RDF and integrated RDF of Al-oxygen distances inAl-Aβ16


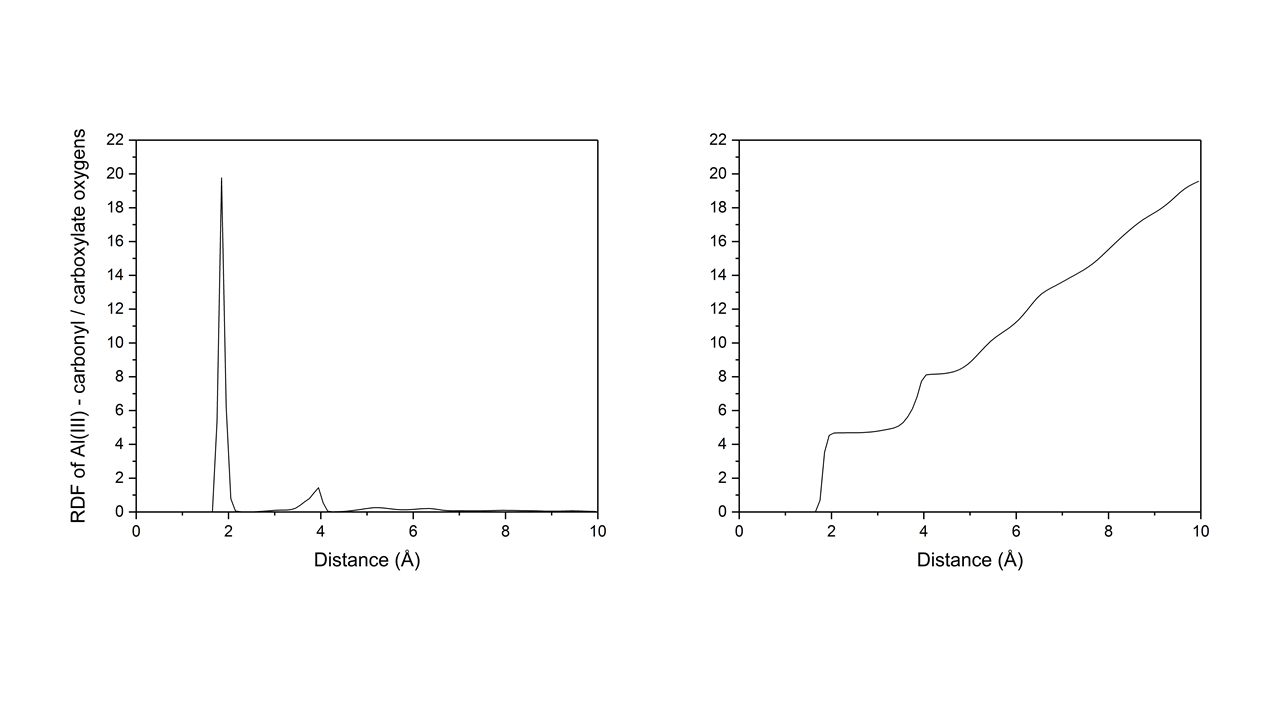

Supplement: S1 Fig — (DOCX) [file pone.0217992.s001.docx]

S2 Figure: RDF and integrated RDF of Al-oxygen distances inAl-Aβ40


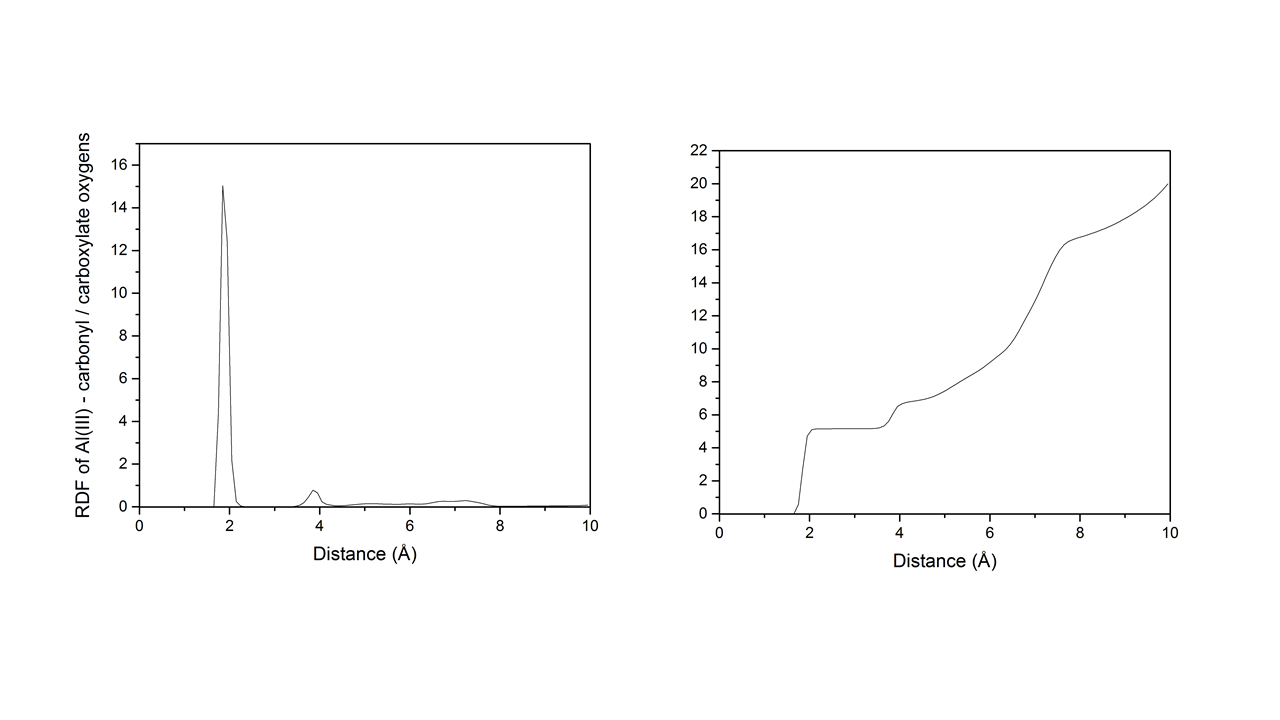

Supplement: S2 Fig — (DOCX) [file pone.0217992.s002.docx]
