# Supplementary material for: Molecular dynamics simulation of aluminium binding to amyloid-β and its effect on peptide structure
Source: PLoS One. 2019 Jun 11;14(6):e0217992. doi: 10.1371/journal.pone.0217992 (PMC6559712; doi:10.1371/journal.pone.0217992)
Supplement: S1 Table — (DOCX) [file pone.0217992.s003.docx]

S1 Table: RMSD data for individual MD runs

| **Al-AB16** | | **Ave** | | **SD** | | **Min** | | **Max** | |
| --- | --- | --- | --- | --- | --- | --- | --- | --- | --- |
| A | | 2.609 | | 0.452 | | 0.846 | | 5.070 | |
| B | | 2.630 | | 0.471 | | 0.991 | | 5.148 | |
| C | | 4.329 | | 0.486 | | 2.584 | | 6.589 | |
| **Al-AB40** | | **Ave** | | **SD** | | **Min** | | **Max** | |
| A | | 8.181 | | 1.016 | | 2.921 | | 12.734 | |
| B | | 9.087 | | 0.865 | | 3.011 | | 11.977 | |
| C | | 8.001 | | 0.799 | | 2.979 | | 11.607 | |
| D | | 8.853 | | 0.896 | | 3.097 | | 13.242 | |
| E | | 8.379 | | 1.178 | | 3.024 | | 14.596 | |
| **Al-AB42** | **Ave** | | **SD** | | **Min** | | **Max** | |  |
| A | 9.778 | | 0.885 | | 6.616 | | 15.294 | |  |
| B | 9.138 | | 0.487 | | 6.980 | | 11.660 | |  |
| C | 9.696 | | 1.054 | | 6.521 | | 16.465 | |  |
| D | 8.588 | | 0.435 | | 7.169 | | 10.899 | |  |
| E | 9.855 | | 0.828 | | 6.690 | | 16.151 | |  |
