# Supplementary material for: Molecular dynamics simulation of aluminium binding to amyloid-β and its effect on peptide structure
Source: PLoS One. 2019 Jun 11;14(6):e0217992. doi: 10.1371/journal.pone.0217992 (PMC6559712; doi:10.1371/journal.pone.0217992)
Supplement: S2 Table — (DOCX) [file pone.0217992.s004.docx]

S2 Table: RMSF by residue

| Residue | 16 | 40 | 42 |
| --- | --- | --- | --- |
| 1 | 4.2715 | 5.7996 | 9.3434 |
| 2 | 2.447 | 3.8733 | 6.9279 |
| **3** | **3.2336** | **2.5075** | **4.5819** |
| 4 | 2.7048 | 2.9664 | 5.7536 |
| 5 | 3.5974 | 3.5683 | 8.5801 |
| 6 | 2.8285 | 3.9775 | 6.5631 |
| **7** | **2.7189** | **2.6402** | **4.4513** |
| 8 | 2.1972 | 2.7842 | 5.9812 |
| 9 | 3.0406 | 3.3164 | 4.0442 |
| 10 | 3.2343 | 4.1911 | 6.0929 |
| **11** | **2.1083** | **1.8764** | **4.0768** |
| 12 | 2.4047 | 3.0365 | 4.0306 |
| 13 | 4.2453 | 3.9753 | 5.0734 |
| 14 | 4.1018 | 3.5988 | 5.787 |
| 15 | 4.1141 | 3.8315 | 5.082 |
| 16 | 5.7345 | 4.849 | 4.6136 |
| 17 |  | 5.062 | 6.1272 |
| 18 |  | 3.8958 | 6.4639 |
| 19 |  | 4.075 | 6.2788 |
| 20 |  | 4.1016 | 6.2861 |
| 21 |  | 3.3753 | 5.4186 |
| 22 |  | 2.9862 | 7.105 |
| 23 |  | 3.6841 | 7.2391 |
| 24 |  | 3.5721 | 8.1749 |
| 25 |  | 2.9488 | 7.9957 |
| 26 |  | 3.3546 | 8.548 |
| 27 |  | 4.1918 | 7.6567 |
| 28 |  | 4.225 | 6.7462 |
| 29 |  | 4.1742 | 6.874 |
| 30 |  | 4.9086 | 6.4706 |
| 31 |  | 5.2053 | 6.1271 |
| 32 |  | 5.2298 | 5.5055 |
| 33 |  | 3.4219 | 4.2954 |
| 34 |  | 3.7097 | 5.3015 |
| 35 |  | 4.9975 | 6.7486 |
| 36 |  | 4.7228 | 7.5844 |
| 37 |  | 5.2265 | 7.7805 |
| 38 |  | 6.4708 | 7.7959 |
| 39 |  | 7.5303 | 8.4346 |
| 40 |  | 8.6539 | 8.4037 |
| 41 |  |  | 9.7756 |
| 42 |  |  | 11.8034 |
