# Supplementary material for: Molecular dynamics simulation of aluminium binding to amyloid-β and its effect on peptide structure
Source: PLoS One. 2019 Jun 11;14(6):e0217992. doi: 10.1371/journal.pone.0217992 (PMC6559712; doi:10.1371/journal.pone.0217992)
Supplement: S3 Table — (DOCX) [file pone.0217992.s005.docx]

S3 Table: Salt bridge incidences (%)

| **AΒ16 SB %** | **Arg5** | **Lys16** |
| --- | --- | --- |
| **Asp1** | 53.94 | 12.10 |
| **Glu3** | 0.00 | 2.12 |
| **Asp7** | 0.00 | 0.10 |
| **Glu11** | 19.72 | 15.67 |

| **Aβ40 SB %** | **Arg5** | **Lys16** | **Lys28** |
| --- | --- | --- | --- |
| **Asp1** | 31.79 | 0.26 | 5.05 |
| **Glu3** | 0.00 | 0.11 | 0.00 |
| **Asp7** | 1.20 | 24.43 | 12.62 |
| **Glu11** | 0.00 | 0.00 | 0.00 |
| **Glu22** | 26.91 | 6.70 | 25.95 |
| **Asp23** | 50.63 | 24.14 | 26.71 |

| **Aβ42 SB %** | **Arg5** | **Lys16** | **Lys28** |
| --- | --- | --- | --- |
| **Asp1** | 42.71 | 0.03 | 0.00 |
| **Glu3** | 0.00 | 0.00 | 0.00 |
| **Asp7** | 0.00 | 0.00 | 0.00 |
| **Glu11** | 0.00 | 0.00 | 0.00 |
| **Glu22** | 13.53 | 7.93 | 14.91 |
| **Asp23** | 8.17 | 26.60 | 37.19 |
